# Supplementary material for: Time-on-task-related decrements in performance in the rodent continuous performance test are not caused by physical disengagement from the task
Source: NPP Digit Psychiatry Neurosci. 2025 Feb 13;3:4. doi: 10.1038/s44277-025-00025-0 (PMC11825365; doi:10.1038/s44277-025-00025-0)
Supplement: Supplementary file 1 — Supplemental Material [file 44277_2025_25_MOESM1_ESM.docx]

**Supplemental Material**

**Time-on-task-related decrements in performance in the rodent continuous performance test are not caused by physical disengagement from the task**

**Ye Li^1^, Thomas van Kralingen^1^, Megan Masi^1,2^, Brandon Villanueva Sanchez^1,3^, Beyonca Mitchell^1,4^, Joshua Johnson^1,5^, Jorge Miranda-Barrientos^1^, Jason J. Rehg^1^, Keri Martinowich^1,5*^, and Gregory V. Carr^1,2*^**

^1^Lieber Institute for Brain Development, Johns Hopkins University Medical Campus, Baltimore, MD, USA.

^2^Department of Pharmacology and Molecular Sciences, Johns Hopkins University School of Medicine, Baltimore, MD, USA.

^3^University of Nebraska Omaha, Omaha, NE, USA.

^4^Georgia Institute of Technology, Atlanta, GA, USA.

^5^Prairie View A&M University, Prairie View, TX, USA

^5^Department of Psychiatry and Behavioral Sciences and Solomon H. Snyder Department of Neuroscience, Johns Hopkins University School of Medicine, Baltimore, MD, USA

*** Correspondence:**

Keri Martinowich

Suite 300

855 North Wolfe Street

Baltimore, MD 21205

keri.martinowich@libd.org

Gregory V. Carr
Suite 300
855 North Wolfe Street
Baltimore, MD 21205
[greg.carr@libd.org](mailto:greg.carr@libd.org)

**Supplemental Figures and Tables**

**
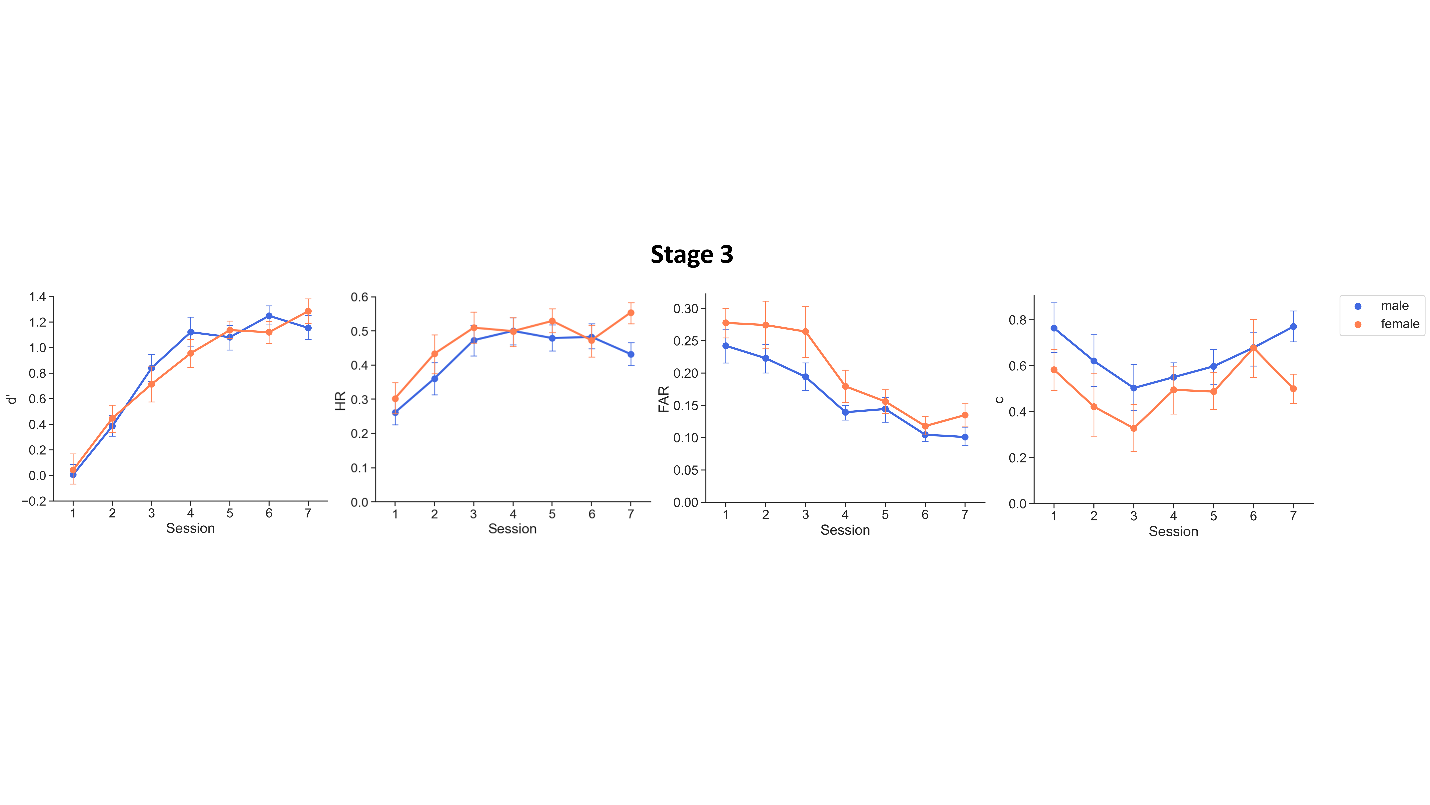
**

**Supplemental Figure 1. No sex differences in performance during Stage 3 training.** Both male and female mice improve performance as measured by d’ across Stage 3 training sessions. The HR increases and the FAR decreases across sessions. Data are shown as mean ± SEM. n = 21 males and 11 females.

**
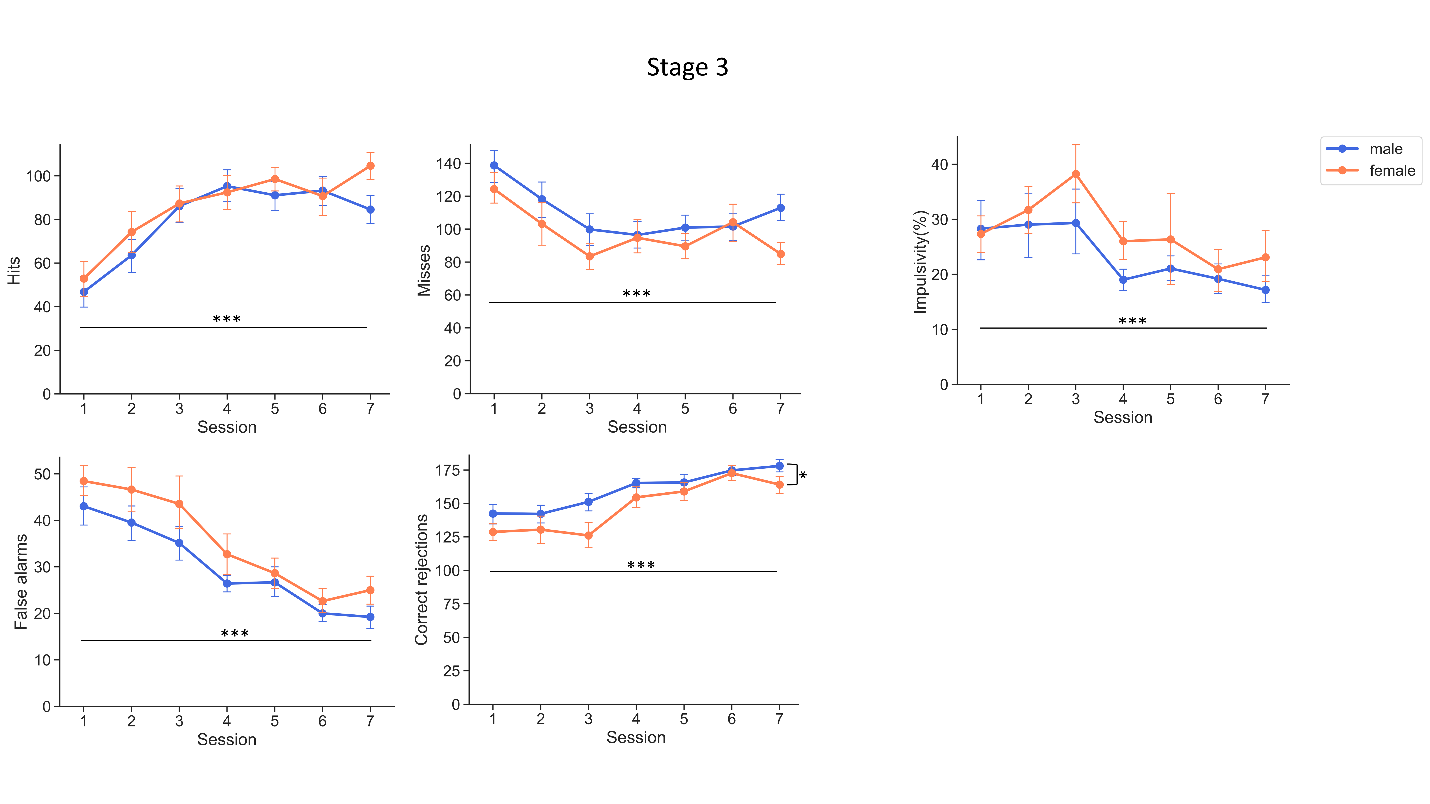
Supplemental Figure 2. rCPT performance during Stage 3 training.** Raw counts for each of the four possible rCPT responses and the impulsivity metric. Data are shown as mean ± SEM. n = 21 males and 11 females. An asterisk (*) indicates statistical significance of main effect of session or sex. *p<0.05, **p<0.01, ***p<0.001**.**

**
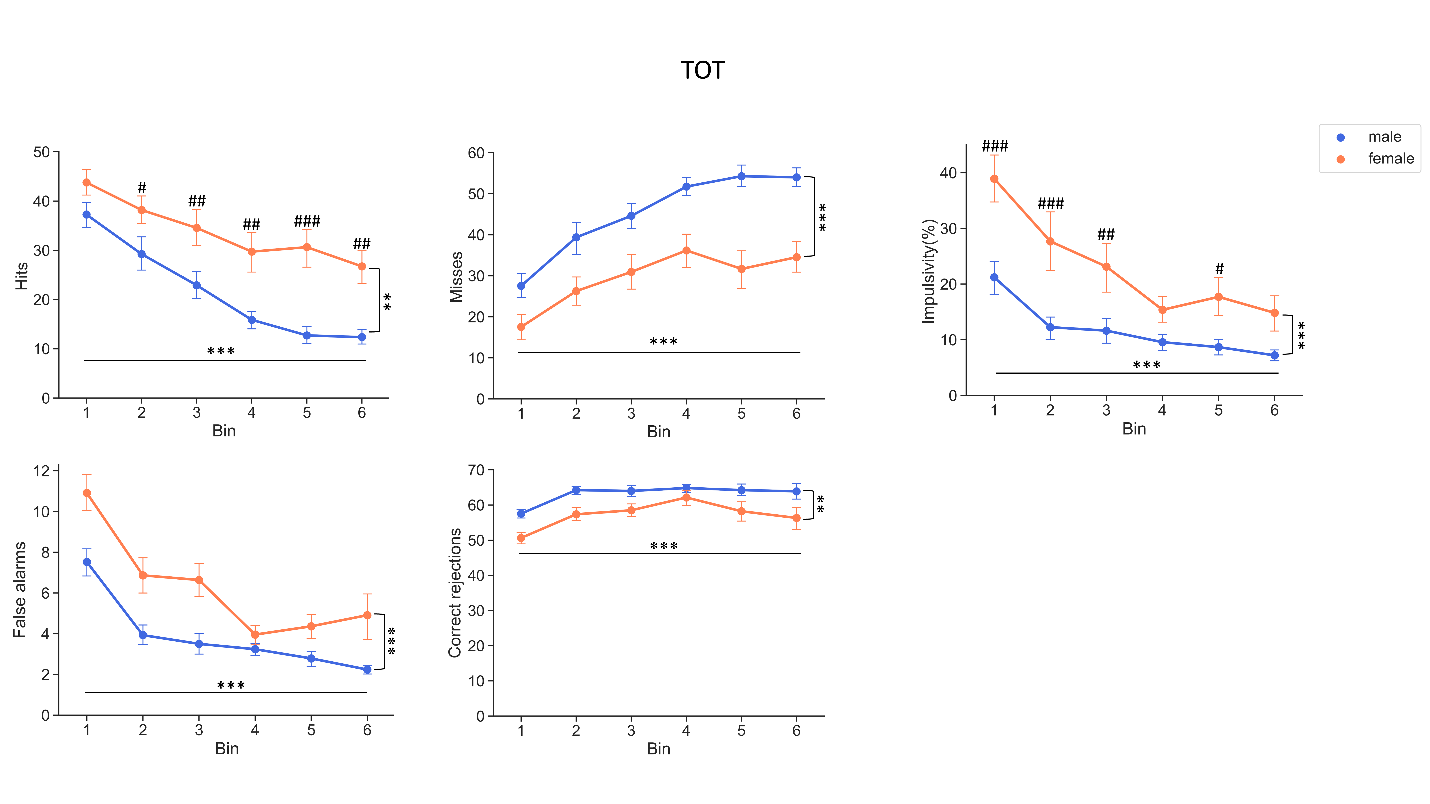
**

**Supplemental Figure 3. rCPT performance during TOT probe trials.** TOT probe sessions were divided into six equal time bins of 15 minutes. Data are shown as mean ± SEM. n = 21 males and 11 females. An asterisk (*) indicates statistical significance of main effect of time bin or sex. A hash (#) indicates significant pos hoc results of time bin X sex interaction. *p<0.05, ***p*<0.01, ****p*<0.001, #*p*<0.05, ##*p*<0.01, ###*p*<0.001​.

**
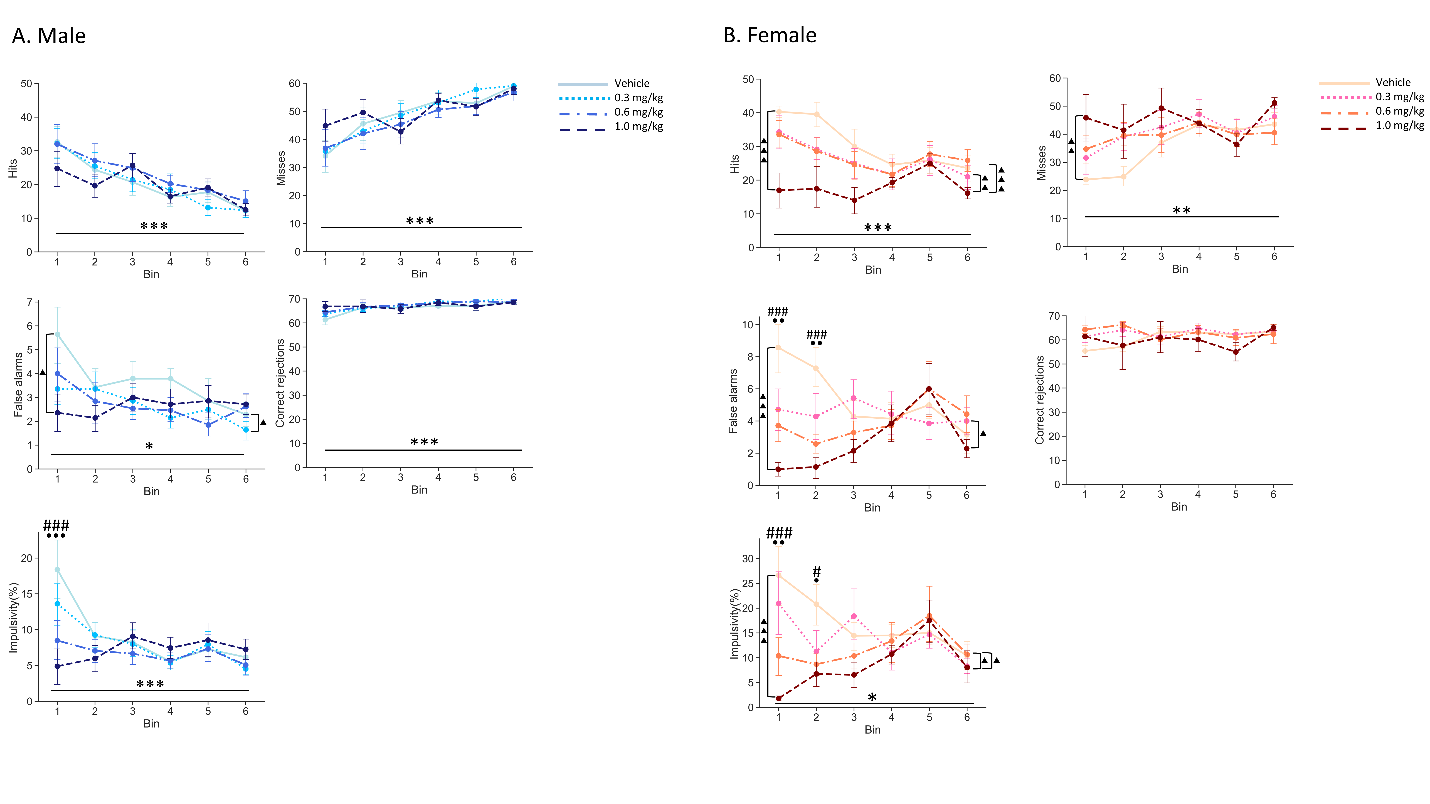
**

**Supplemental Figure 4. Effects of AMPH on rCPT performance. A.** Male **B.** Female. An asterisk (*) indicates statistical significance of main effect or time bin or drug. A triangle (▲) indicates significant post hoc result. A circle (•) indicates the significant effect of 0.6 mg/kg and a hash (#) indicates significant effect of 1 mg/kg after post hoc results of time bin X drug interaction. Data are shown as mean ± SEM. n = 15 males and 7 females. *p<0.05, ***p*<0.01, ****p*<0.001, • *p*<0.05, •• *p*<0.01 , ^##^*p*<0.01, ^###^*p*<0.001.

**
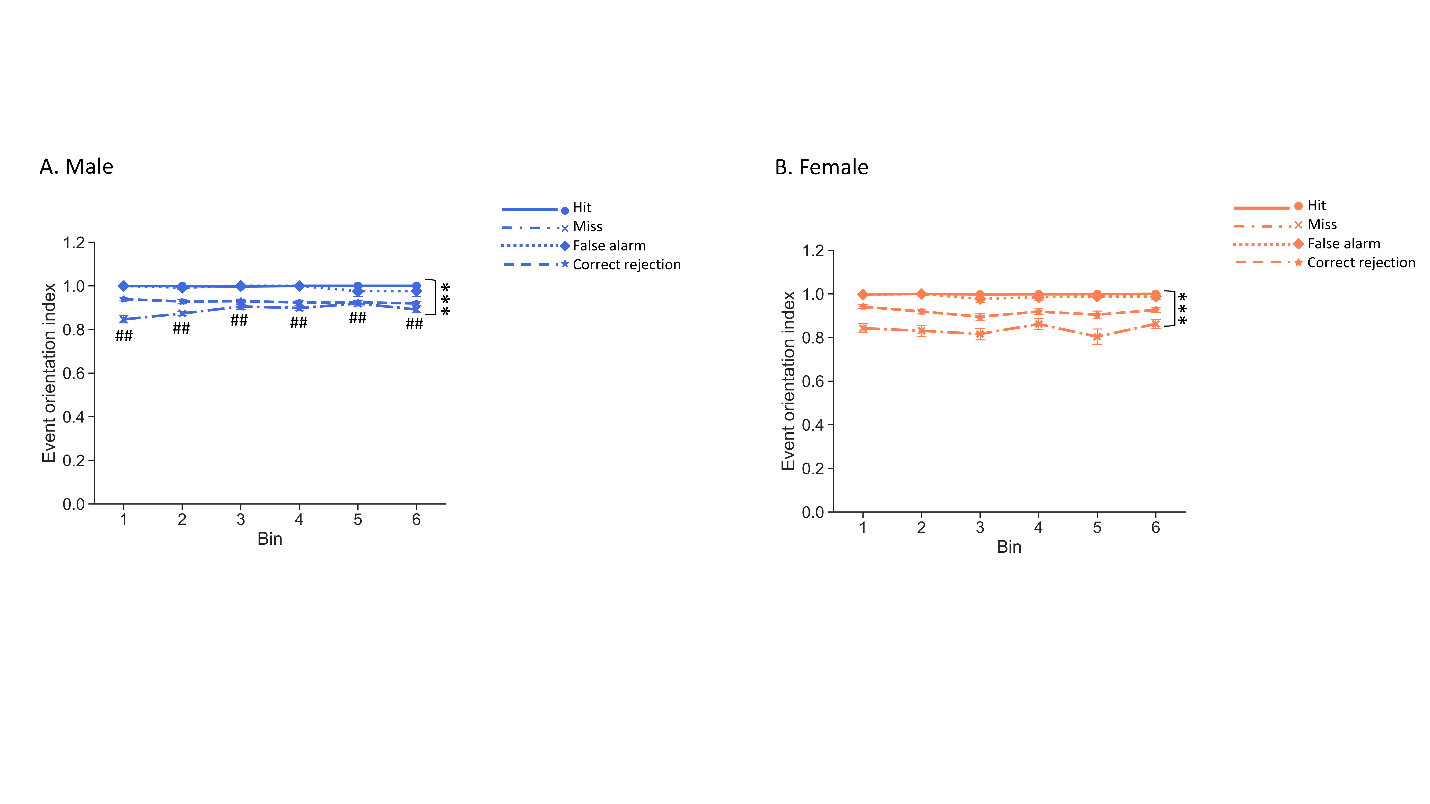
**

**Supplemental Figure 5. Task engagement during TOT probe sessions. A.** Males **B.** Females Data are shown as mean ± SEM. n = 21 males and 11 females. An asterisk (*) indicates statistical significance of main effect of response type. A hash (#) indicates significant post hoc results of time bin X response type interaction. ****p*<0.001, ##*p*<0.01.

**
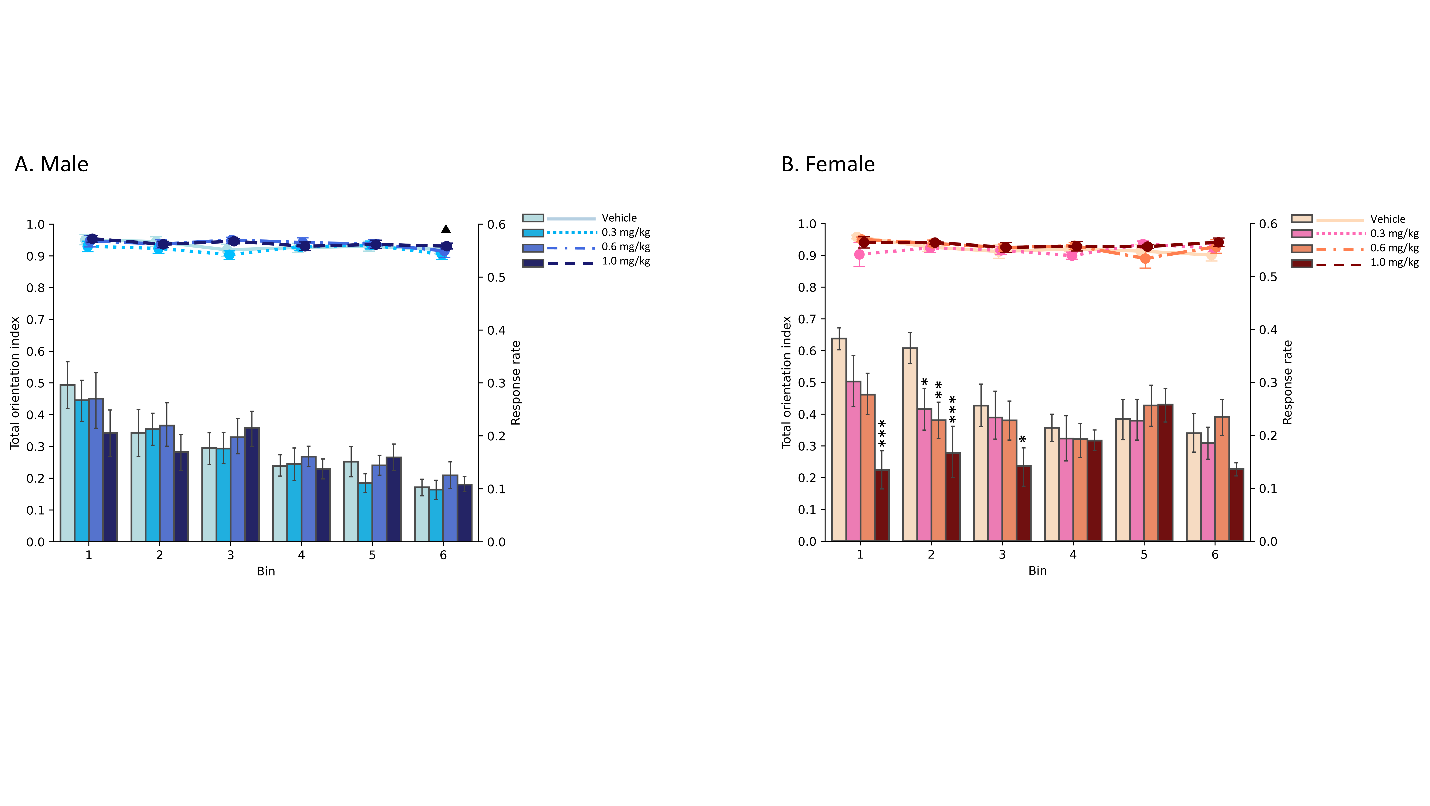
**

**Supplemental Figure 6. AMPH does not affect the orientation index during the limited hold period.** The line plot presents the total orientation index. The bar plot presents the response rate. **A** The orientation index and response rate of male mice after amphetamine administration. AMPH did not alter the orientation index or response rate. **B** The orientation index and response rate of female mice after amphetamine administration. AMPH did not affect the orientation index in female mice. However, AMPH significantly decreased the response rate of female mice. Data are shown as mean ± SEM. n = 15 males and 7 females. A triangle (▲) in total orientation index curve indicates significant post hoc results comparing to bin 1. An asterisk (*****) in response rate bar indicates significant post hoc results comparing to vehicle. **p*<0.05, ***p*<0.01, ****p*<0.001, ^▲^*p*<0.05.

**
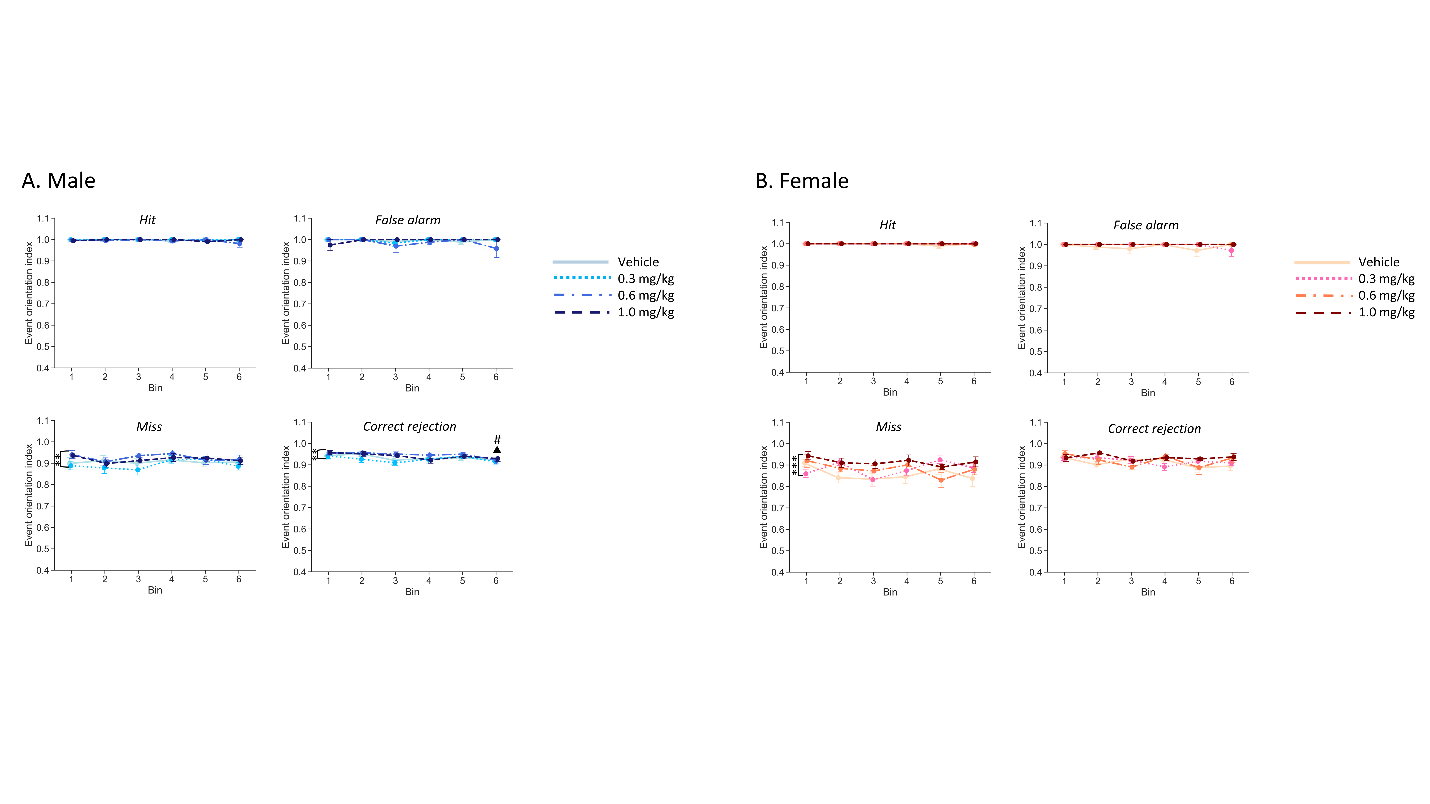
**

**Supplemental Figure 7. AMPH effects on the orientation indices during the limited hold period. A.** Males **B.** Females. Data are shown as mean ± SEM. n = 15 males and 7 females. A main effect of time bin where there was a decrease in orientation during correct rejection trials between Bin 1 and Bin 6 (indicated by ▲)*.* B The orientation index of female mice after amphetamine administration. Main effect of drug was shown in orientation index of miss (indicated by *). **p*<0.05, ^▲^p<0.05..

**
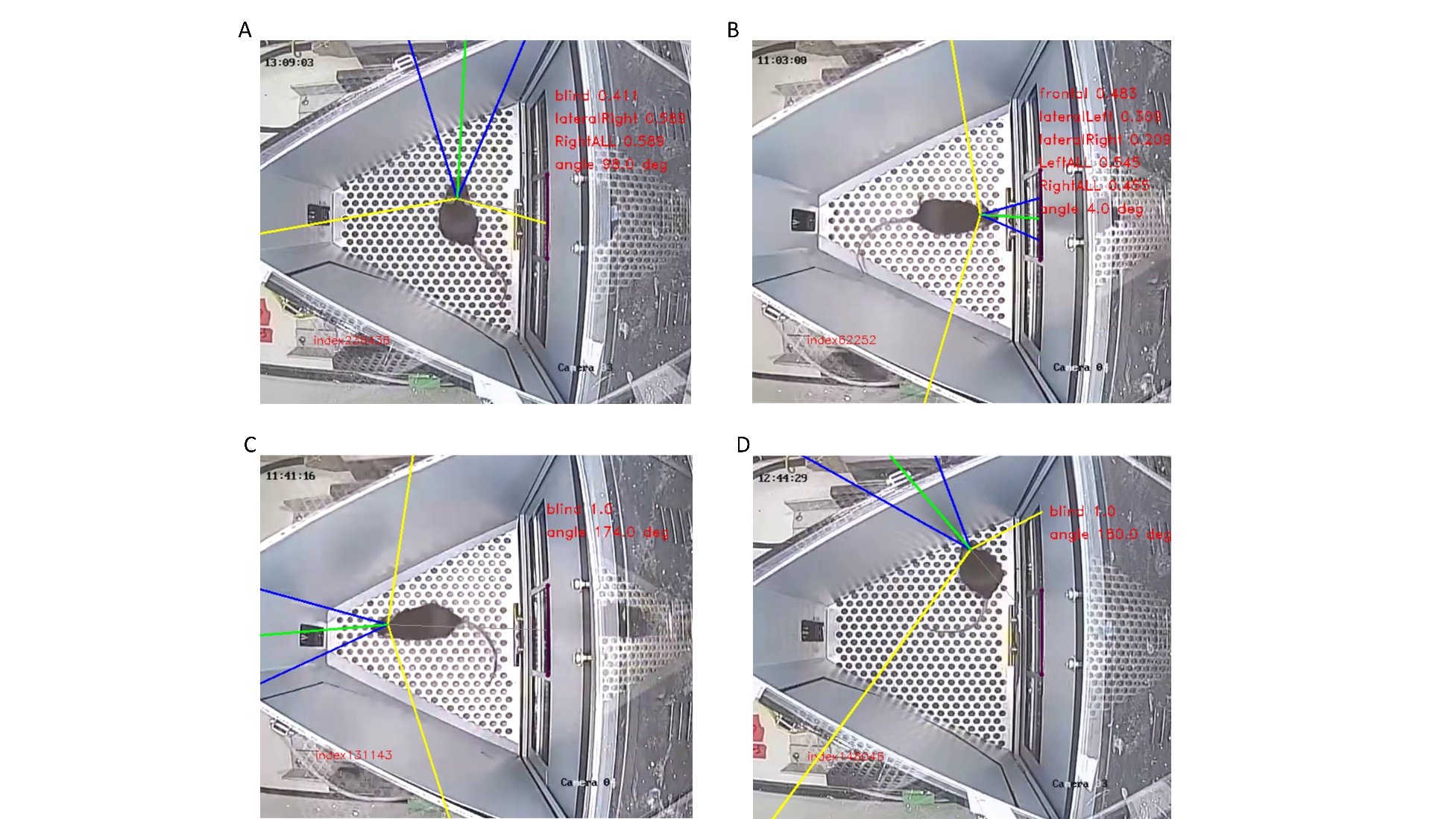
**

**Supplemental Figure 8. Example of VFA results plot. A, B** Mouse oriented toward the screen. **C, D** Mouse blinded to the screen. The area between the green and yellow lines represents the entire right or left visual field, while the acute angle between the two blue lines defines the frontal visual area. The region between the two yellow lines corresponds to the blind area (behind the ears), and the purple line indicates the stimulus location on the touchscreen. The value following each area name represents the proportion of the stimulus within that visual area. For example, in panel A, .411 of the stimulus lies within the blind area, while .589 lies within the right-side visual area.


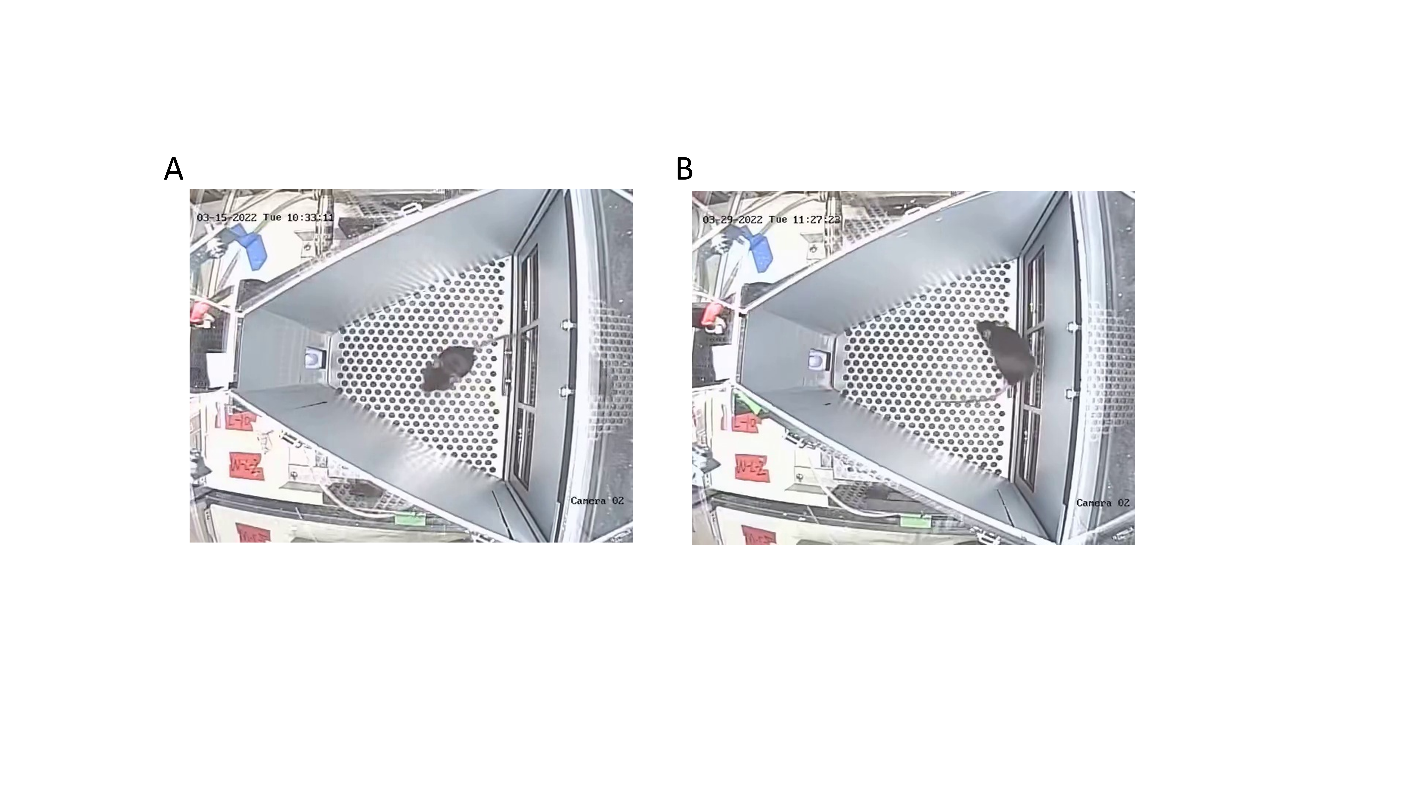


**Supplemental Figure 9. Example of unoriented hit even. A** mouse touched the screen by using the tail. **B** mouse touched the screen by using the paw.

**Supplemental Table 1**


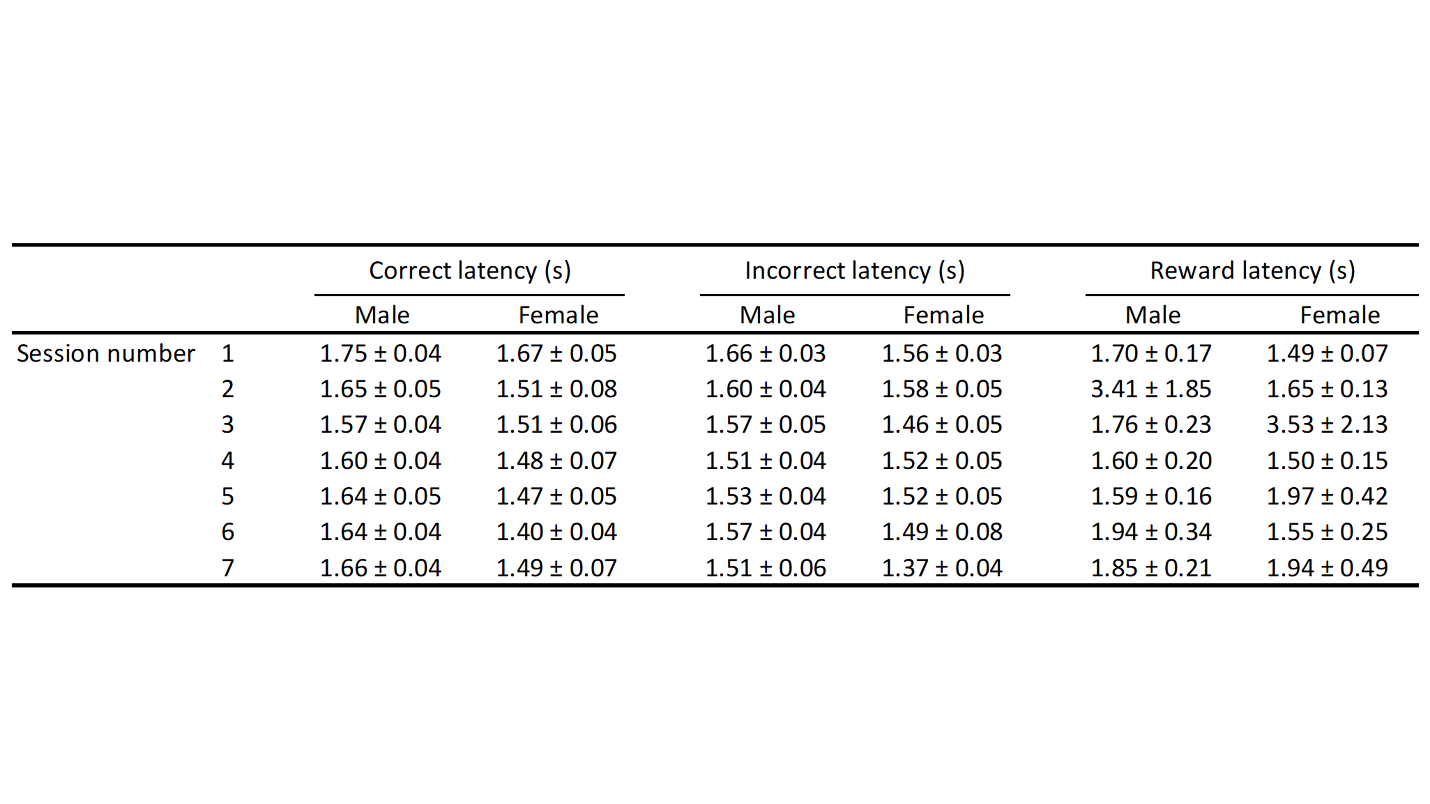


**Supplemental Table 2**

**
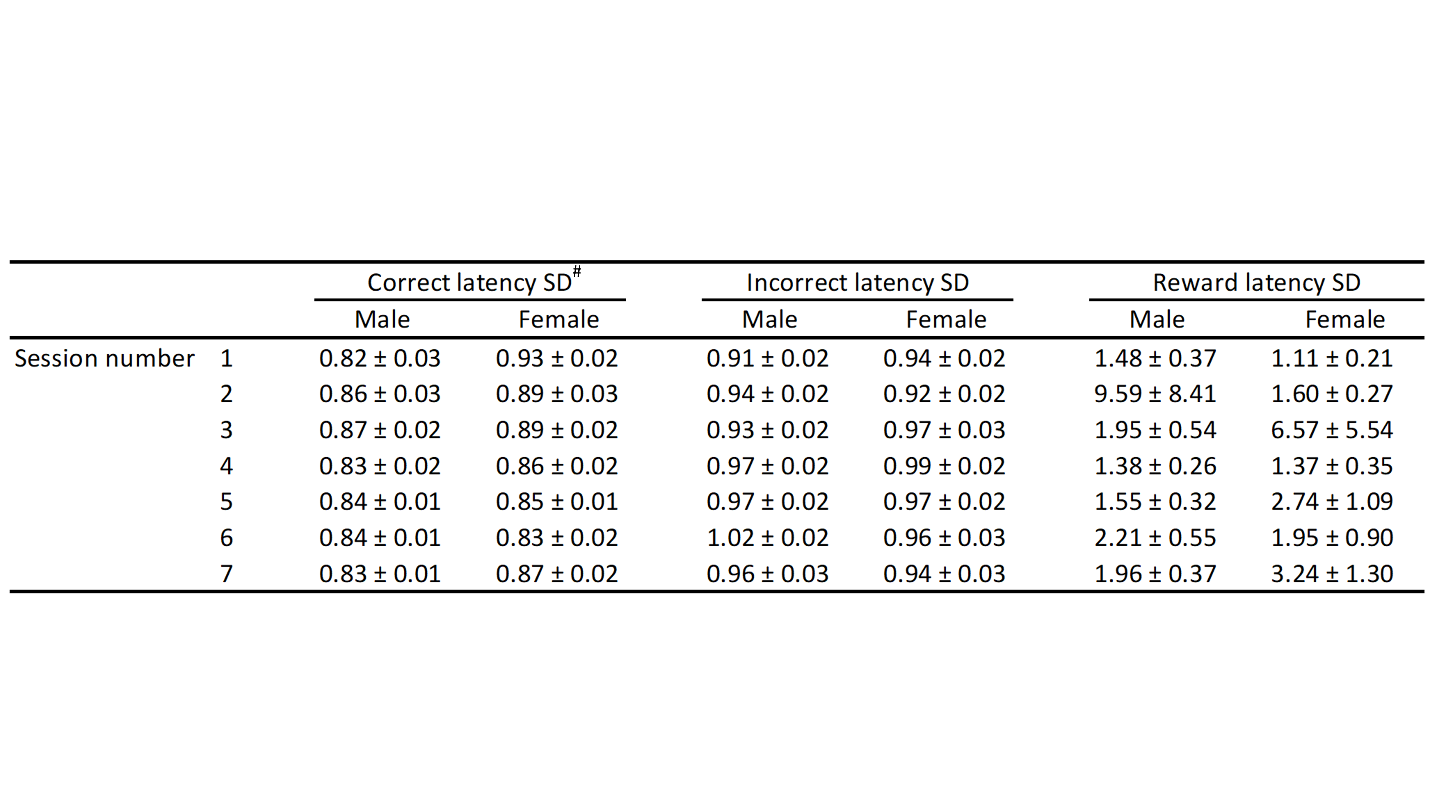
**

**Supplemental Table 3**

**
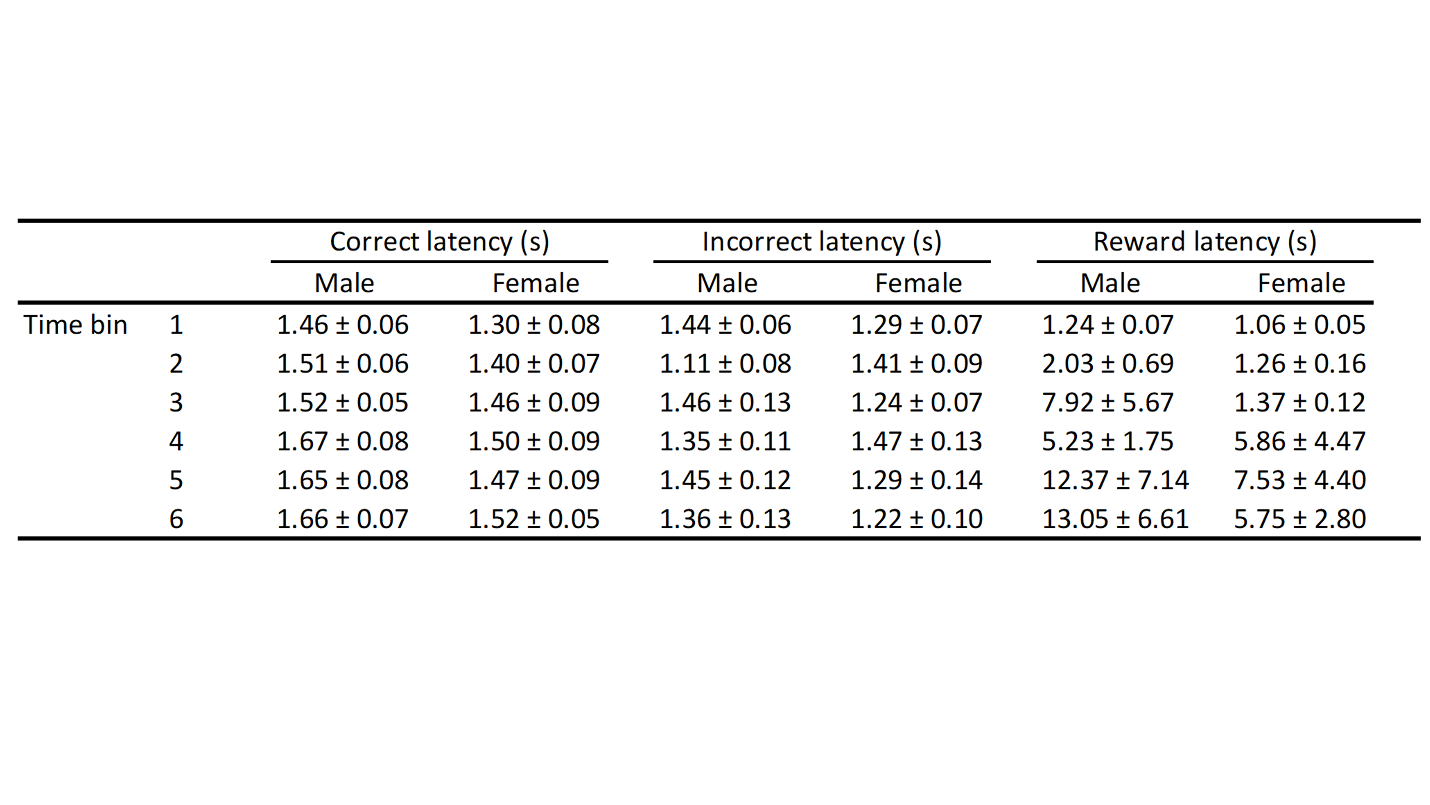
**

**Supplemental Table 4**

**
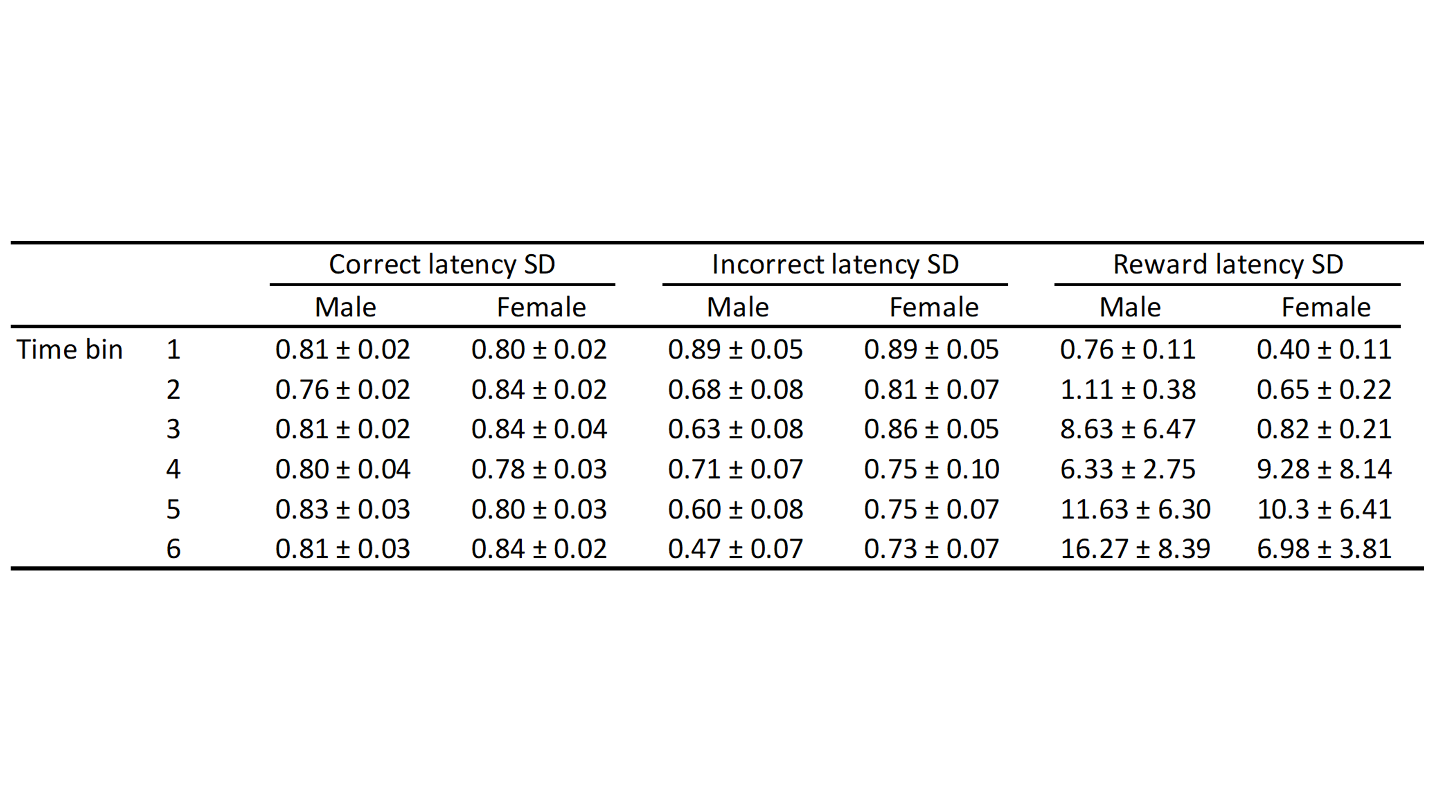
**

**Supplemental Table 5**

**
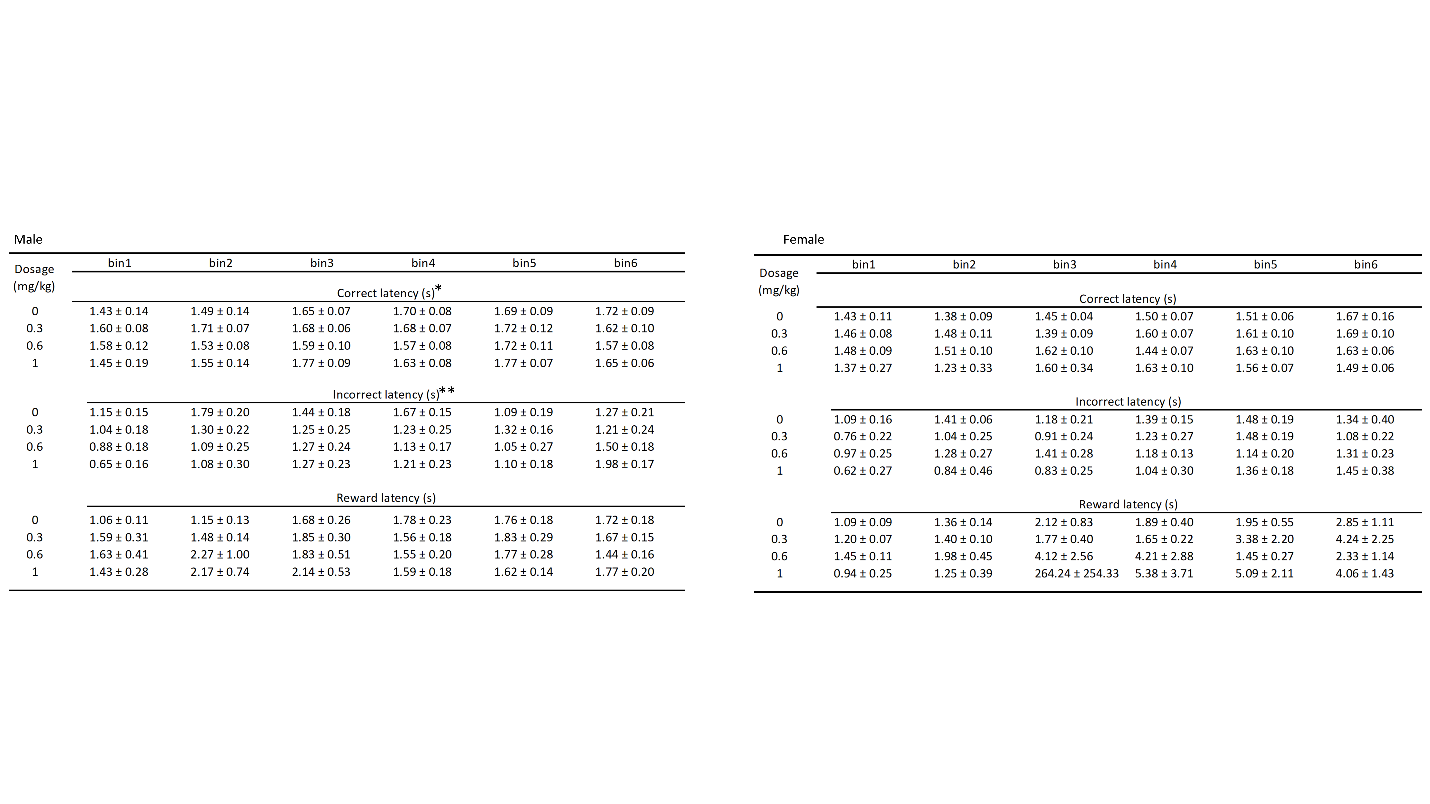
**

**Supplemental Table 6**

**
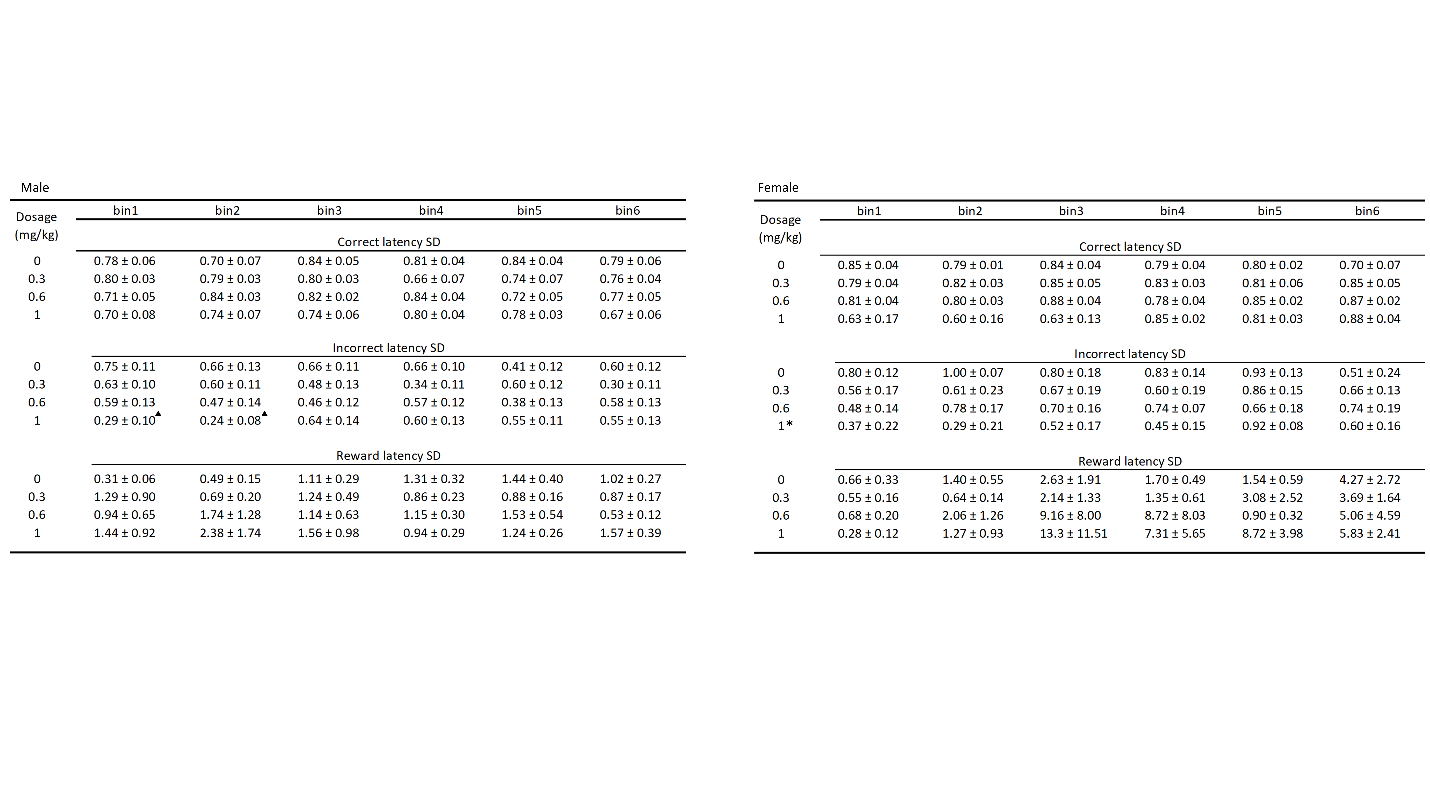
**
